# Multi-Scale Spatial Prediction of Wild Boar Damage Risk in Hunchun: A Key Tiger Range in China

**DOI:** 10.3390/ani11041012

**Published:** 2021-04-03

**Authors:** Yongchao Jin, Weiyao Kong, Hong Yan, Guangdao Bao, Ting Liu, Qiongfang Ma, Xinhai Li, Hongfei Zou, Minghai Zhang

**Affiliations:** 1 College of Wildlife and Protected Areas, Northeast Forestry University, Harbin 150040, China; ychjin@wwfchina.org (Y.J.); hongfeizou@163.com (H.Z.); 2 World Wild Fund for Nature (China), Changchun 130022, China; 3 Jilin Provincial Academy of Forestry Science, Changchun 130033, China; kongweiyao@163.com (W.K.); yanh458@163.com (H.Y.); bao-gd@126.com (G.B.); liutingcn@outlook.com (T.L.); youzi841128@163.com (Q.M.); 4 Jilin Momoge Wetland Ecosystem Research Station, Changchun 130033, China; 5 Jilin Provincial Key Laboratory of Wildlife and Biodiversity in Changbai Mountain, Changchun 130033, China; 6 Institute of Zoology, Chinese Academy of Sciences, Beijing 100101, China; lixh@ioz.ac.cn

**Keywords:** wild boar damage, multi-scale, risk prediction, Maximum Entropy Model, species-specific model tuning

## Abstract

**Simple Summary:**

Spatial distribution of wild boar damage risk is important and can be informative to wildlife habitat management. Hunchun is an important active area of Siberian tiger in China. The wild boar damage has brought barriers to the conservation and management of the Siberian tiger in this region. We predicted the spatial distribution of wild boar damage risk in Hunchun in terms of home range and feeding sites scales, and explored the spatial interaction between tiger habitats and the damage risk of wild boar. The results show the distance to the forest edge is an important factor affecting the wild boar damage, and 38.68% of the high-risk areas are overlapped with tiger habitats in Hunchun. Therefore, precise and differentiated management strategies should be adopted in the management of wild boar population.

**Abstract:**

Hunchun, a typical area suffering wild boar (*Sus scrofa*) damage, is an important region for the Siberian Tiger (*Panthera tigris*) in China. By incorporating the maximum entropy model with 22 variables in the home range scale (12 variables) and in the feeding site scale (10 variables), we predicted wild boar damage risks in this area of China and analyzed how spatial factors influence damage risk. Damage risk was found to be high in areas close to the forest edge, areas with a higher forest cover and lower to medium deciduous forest proportion, low road density, and a medium river density and farmland proportion. The proportion of farmland which was identified as being in the high damage risk zone was 23.55%, of which 38.68% was within the habitat area of the Siberian Tiger. Finally, we propose wild boar damage prevention based on different management goals.

## 1. Introduction

Globally, issues relating to damage due to wild boars (*Sus scrofa*) have become significant areas for wildlife management [1,2,3,4]. Crop damage has become the most important issue [5,6,7] along with other areas of concern including the spread of infectious diseases [8,9,10], gene contamination of domestic pigs, grassland rooting [2], and direct danger to local settlements [11]. Management practices to control crop damage include wild boar hunting [12,13], providing available food resources in the forest [12,14], erecting fences to exclude the animals [15], using chemical repellents [16], and positioning devices to frighten away the animals [17].

Spatial pattern, a very important factor in human–wild boar conflict management, provides basic information for management strategies, and it aids in explaining how spatial factors influence damage occurrence mechanisms [4,18]. Wildlife distribution is an inherently scale-sensitive process with animals often selecting environment resources at different levels [19]. Previous studies have examined various factors influencing wild boar damage to crops, including population density, road density, forest fragmentation, land-use type [2,18], shelter structure [20], forest type, soil humidity [4], and distance to river, road, and forest edge [21,22]. To date, the majority of studies relating to wild boar damage have used a single spatial scale; studies involving multiple spatial scales require further investigation.

Hunchun County is an area suffering wild boar damage. Since the introduction of the hunting ban in 1996, wild boar populations have dramatically increased, coinciding with an increase in crop damage, annual wild boar damage in this area now exceeds 3 million RMB [11]. Wild boars are sympatric with Siberian tiger (*Panthera tigris*), which and Siberian tiger is a representative species for population protection and recovery in China [23]. Approximately 90% of the tiger population in China occurs in the Hunchun region [23,24]. Crop cultivation is an important source of income for households in the area, and farmland can be a barrier for tigers moving from the Russia–China border farther inland [23]. As wild boars are the preferred prey for tigers [25], it is essential that wild boar populations are carefully managed through the analysis of crop damage and that conservation and recovery plans for tigers are developed based on wild boar damage risk.

## 2. Materials and Methods

### 2.1. Study Area

Hunchun County, Jilin Province, covers an area of 5154 km^2^ in the northeast of China, adjacent to Russia and North Korea. This region is characterized by a temperate maritime monsoon climate, with an average annual temperature of 5.6 °C and precipitation of 617.9 mm. The area of forest cover in Hunchun is 84.79%, of which deciduous, mixed coniferous, and coniferous forest account for 30.90%, 49.81%, and 19.29% of forested land, respectively. Forest border areas are embedded within an agricultural matrix, predominantly dominated by corn and rice (11.75% of land cover).

Hunchun County contains the greatest diversity of fauna in the northern forest ecosystem of China, with very high abundances of wild boar, sika deer (*Cervus nippon*), and roe deer (*Capreolus capreolus*). This high abundance of prey provides a suitable habitat for carnivores such as Siberian tigers, leopards (*Panthera pardus*), black bears (*Ursus thibetanus*), and brown bears (*Ursus arctos*).

### 2.2. Data Collection

A total of 357 cases of wild boar damage were recorded from wildlife compensation application files in Hunchun between 2014 and 2015. All of the cases of wild boar damage were recorded in farmland. Snow survey records (19), camera trap events (296), and cases of cattle being killed by tiger attacks (191) provided information regarding tiger presence in this area.

Land-use type (coniferous forest, mixed coniferous-deciduous forest, deciduous forest, farmland, settlements, water, road, and other land-use) was extracted from GF-2 remote sensing images (acquisition time: September 2015, resolution: 0.8 m) by manual visual interpretation. Rivers and roads were also visually extracted from the image. The digital elevation model was downloaded from ASTER GDEM (http://gdem.ersdac.jspacesystems.or.jp/, accessed on 15 March 2017).

We used a 30 × 30 m grid to examine 22 variables influencing the home range and feeding site scales [26]. A 30 × 30 m grid is the minimum area accepted for crop damage compensation. As per Keuling [27] and Ficetola [18], we defined the home range scale as a 2 km-radius circle around the central point of the grid and the feeding site scale as the central point of the grid. Twelve factors were extracted in the home range scale: landscape diversity (number of land-use types), farmland fragmentation (perimeter/area), proportion of forest, deciduous forest, mixed forest, coniferous forest, farmland and settlements, road and water density (km/km^2^), average elevation, and slope. For the feeding site scale, we extracted ten factors: elevation, slope, aspect, distance to forest edge, deciduous forest, mixed forest, coniferous forest, settlements, water, and road.

### 2.3. Data Analysis

Environmental layer data were standardized and performed with coefficient analysis, and we used the maximum entropy model to predict wild boar damage risk. The model used the distribution of occurrence records to estimate a target probability distribution of wild boar damage. In order to eliminate spatial autocorrelation, we used masked geographically structured evaluations [28] to partition data to four bins and performed k-fold cross-validation. The k-1 bins were used for model training and the withheld bin for testing. Response curves were used to identify the effects of variables on prediction results. Models with regularization multiplier (RM) values ranging from 0.5 to 6.0 (increments of 0.5) and with five different feature class (FC) combinations (L, LQ, H, LQH, LQHP; where L = linear, Q = quadratic, H = hinge, and P = product) were also constructed [29]. Of the 60 models constructed for this study, we chose the model having the minimum delta Akaike information criterion corrected for sample size (delta.AICc) as the optimal one for prediction [29,30].

We used a maximum training sensitivity sum specificity threshold to identify high damage risk zones [31]. The area of the high damage risk zone was calculated by summing up the grids with predicted values higher than the threshold.

We used records of tiger presence to identify the distribution of tigers using the kernel density estimation with a 95% volume contour (95% KDE contour). We then extracted the high wild boar damage risk zone within the 95% KDE contour and calculated the area.

Environmental variables extraction, layer projection, and area calculation were performed using ArcGIS 10.2. Data partitions and model selections were executed using ENMeval [29], an R package based on the Maxent model. Response curve figures and high-risk threshold evaluation were achieved in dismo 1.1–4 [32]. Fixed Kernel Density Estimation and volume isopleth were undertaken using GME 0.7.3 [33].

## 3. Results

### 3.1. Model Selection

**Figure 1:**
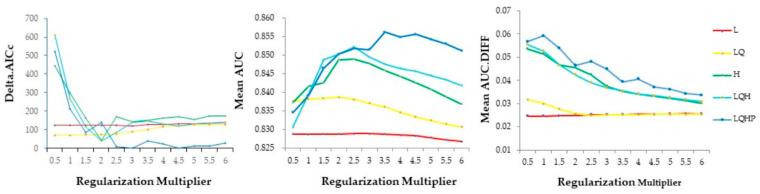
The delta.AICc, mean AUC, and mean AUC.DIFF metrics resulting from Maxent models made across 5 feature class and 12 regularization multiplier combinations. The left panel shows delta.AICc; the center panels show mean AUC, and the right panel shows the mean difference between training and testing (AUC.DIFF) data. L = linear, Q = quadratic, H = hinge, P = product.

The optimal model for our study (Figure 1) had a regularization multiplier of 4.5 and a feature of LQHP (LQHP 4.5 model) showing the lowest delta.AICc (delta.AICc = 0). The mean area under receiver operating characteristic (AUC) of the LQHP 4.5 model was 0.8556, this being slightly lower than the maximum value in the LQHP 3.5 model (0.8561). The mean difference between the training and testing AUC (AUC.DIFF = 0.037) was a comparatively low level, showing a low degree of model overfitting [34].

### 3.2. Factors Influencing Wild Boar Damage Risk

**Figure 2:**
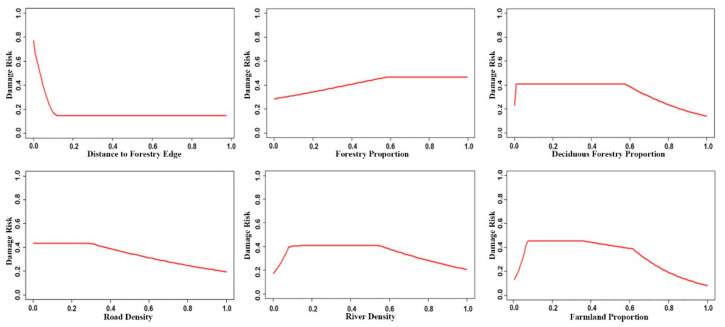
Response curve of the six most important variables contributing to the LQHP 4.5 model. The lateral axis shows the standardized value of environmental variables, and the vertical axis shows the standardized predicted value of wild boar damage risk. The distance to forest edge is the variable of the feeding site scale and others are the home range scale.

Distance to the forest edge and forest proportion were the most important factors influencing wild boar damage, followed by deciduous forest proportion, road density, river density, and farmland proportion. These six factors contributed 86.1% to the LQHP 4.5 model. (Table 1). Response curves of these six factors to damage risk are shown in Figure 2. Distance to the forest edge and road density were negative factors affecting wild boar damage risk, with forest proportion having a positive influence. River density, deciduous forest, and the proportion of farmland recorded a piecewise response pattern. The level of risk was raised at the low value range of those factors, after which it was maintained at a high level in the low to medium values, before finally decreasing with high values.

### 3.3. Spatial Pattern of Wild Boar Damage Risk

**Figure 3:**
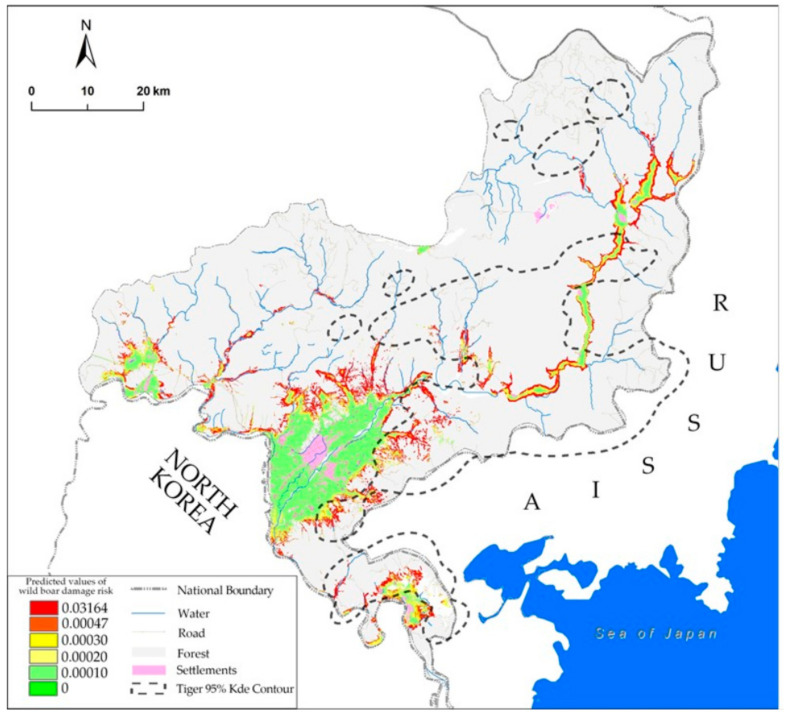
Wild boar damage risk prediction in Hunchun County. The two warmest colors (values above 0.000468) indicate high levels of damage risk. The black dotted line indicates the tiger distribution area within 95% of the kernel density estimation contour.

The maximum training sensitivity sum specificity threshold was 0.000468 (raw output value). Our results from the optimal model showed that 23.55% of farmland was identified as being a zone of high damage risk (Figure 3).

The area of tiger distribution was 1922.8 km^2^ within the 95% KDE contour, and 38.68% of the high wild boar damage risk farmland was located in the tiger distribution area.

## 4. Discussion

The spatial distribution pattern of wildlife damage is one of the most significant references in the management of human wildlife conflicts and is also an important aspect of wildlife conservation [35]. Among all species, wild boars account for the largest number and the biggest cause of all damages and losses in such conflicts [1,4]. In Hunchun, the government spends 3 million RMB each year on managing the damages caused by wild boars. Moreover, Hunchun is also the most concentrated area in China with the largest number of Siberian tigers [23], for which wild boars are their favorite prey [25]. Therefore, it is an issue of emergency to reduce the damages caused by wild boars while keeping their population at a necessary scale to provide enough prey for tigers. In our research, we found that damages by wild boars mainly occurred in farmland, of which 23.55% are at a high risk (Figure 3). This is significantly related to the fact that most of the crops are cash crops, such as corn, and the lack of food resource by human collection of non-timber forest products in the spring and autumn [36]. Therefore, we suggest that croplands in high-risk areas be planted with crops that are not appealing to wild boars [2], such as soybeans (*Glycine max*) and rice (*Oryza sativa*). Accurate monitoring of the wild boar population would also be necessary in these areas to ensure that preventative measures are formulated in correspondence with their population dynamics. In addition, controlling human activities during harvest seasons could also help to reduce wild boar damages by allowing more food resources for them.

In areas with a high risk of wild boar damage, the management of the wild boar population is utilized through different strategies which play an important role in the recovery of the Siberian tiger population. Our study has revealed that 38.68% of the high-risk areas overlap with the areas populated by tigers (Figure 3), which form important gateways among their habitats. Therefore, we suggest that food supply management [14] and forest restoration [35] measures be implemented in order to keep the wild boar population at a relatively sufficient scale. For the food supply management, necessary food such as corn should be supplied throughout the forest, and the food sites should be more than 1 km away from the edge of the forest and extend evenly into forest.

According to the wildlife damage compensation policy, only damage exceeding 0.1 hectare in registered farmland is accepted. The maximum entropy model only uses presence data [37,38], thereby removing issues related to unreliable absence [39]. A fundamental limitation of using presence-only data is that sample selection bias has a much stronger effect than presence-absence models [40,41], and masked geographical structured evaluations provide an effective approach to sidestep spatial bias [28]. The performance of the Maxent model critically depends on the model complex, which calls for specific tuning of FCs and RM combination [37,42]. The Maxent model limits the model complexity and protects against overfitting by RM [38]. The RM of the optimal model was 4.5, indicating that using a higher RM is necessary to achieve optimal model complexity rather than using a default setting [28,38].

Animal distribution is a scale-sensitive and hierarchically structured process [43]. Because habitat selection depends on scale and selection, depending on the ratio of reward to risk, this implies that the ratio of reward to risk should also depend on scale [44]. Johnson [26] defined four orders of habitat selection scales: species range, home range, feeding site, and food items. Home range and feeding sites were previously highlighted as being the most informative scales [45]. As crop damage is a temporal feeding site selection of wild boar, species range is therefore too extensive to analyze crop damage. The food item scale in this study was also not used as corn crops are considered a highly homogeneous crop type since the implementation of the corn storage policy in 2007.

The distance to the forest edge was the most important factor affecting wild boar damage. Although this is a widely recognized conclusion [3,4,5,19,21,23], Ficetola [18] argued against this finding. Damage risk recorded a sharp decrease with increasing distance to the forest edge (Figure 2); the average distance to forest edge was 92 m and 82% of damage occurred within 150 m. This distance was much shorter than the wild boar shift length during the crop harvest season [27]. As animals move further from the forest edge, time and energy costs increase [46], yet they get no extra benefits from the homogeneous farmland. The distance to three different forest types was much less important for damage risk, although they were highly related with distance to the forest edge.

Forest was also an important factor in the home range scale, however the response curve was more subtle. A suitable habitat should contain a mixture of patches that fit the animals’ different needs [47]. Increasing forest proportion provides more resources for escaping while reducing the opportunity of accessing farmland; the suitability supported by good shelter was partly reduced due to an increase in energy costs associated with moving to the feeding site [18]. On the contrary, the response curve recorded a rapid increase as the proportion of deciduous forest increased at the beginning. As deciduous forest is the preferred habitat of wild boars during the summer and fall periods [20], the advantage of a more favorable food source (mainly mast) [48] promotes habitat suitability as well as increasing potential damage risk to crops [22]. The variation of food resources could also explain the risk regression in the area with high deciduous forest proportion—as forests provide sufficient food sources and shelter, crop damage will therefore subsequently decline [14].

The contribution of the farmland proportion was only 3.60%, even though it was highly related with the forest proportion and the deciduous forest proportion. Farmland results in an abundance of food resources yet poor refuge quality. When farmland was a limited resource with a low value, the energy reward played a leading role in forming foraging strategy. However, when forest size became very small, risk avoidance dominated the process [49], and animals chose less productive foraging habitats due to safety concerns [50].

Human disturbance has been identified as having a significantly negative influence on crop damage [2,18,21]. Among the four human disturbance factors (road density, distance to road, settlement proportion, and distance to settlement), only road density was ranked as the important variable with a lower contribution (4.33%). Roads are a negative but unimportant factor [10,21] for wild boar crop damage. Road density was highly related with forest, deciduous forest, farmland, and settlement proportion. The influence of roads may therefore be a combination of these related factors.

Studies by Masayuki [3], Li [4] and Cai [21] indicated water was an important factor related to damage; however, our results indicated that water was not a very important factor in either the home range or on the feeding site scale. These results however did not indicate water resources to be unimportant [20]. Crop damage occurs in the rainy season in the study area. The scattered distribution of bogs and ponds in the forest also provide water resources for wild boar; thus, they are not restricted to staying close to rivers. River density was lower in upstream areas, thus resulting in positive damage risk. High values were recorded in Hunchun and in the Tumen river basins with severe human disturbance located away from the forest, thus it became a negative factor.

## 5. Conclusions

Our research shows precise and differentiated management strategies should be adopted in the management of wild boar population to mitigate damage. Farmers in the high-risk zones should be notified so they can avoid planting cash crops favored by wild boars, and precise population monitoring is also required in those areas to ensure corresponding prevention strategies based on population dynamics. In addition, human collections in harvest seasons should also be restricted to allow more food resources for wild boars. Furthermore, within tiger range, available food supply and forest restoration are recommended to keep a necessary population of wild boar for tigers. Finally, the land use planning by the Hunchun government should be based on the results of wild boar damage risk in our research to reduce the wild boar and human conflicts and promote the tiger population recovery.

## Figures and Tables

**Table 1:** Relative importance of environmental variables in the optimal model.

Variable	Percent Contribution (%)
Distance to forest edge	39.13
Forest proportion	27.80
Deciduous forest proportion	7.55
Road density	4.33
River density	3.67
Farmland proportion	3.60
Slope	3.16
Distance to settlement	2.92
Distance to road	2.75
Mixed forest proportion	1.17
Distance to coniferous forest	1.14
Aspect	0.95
Settlement proportion	0.73
Average elevation	0.59
Distance to mixed forest	0.15
Distance to deciduous forest	0.11
Average slope	0.10
Landscape diversity	0.09
Coniferous forest proportion	0.05
Distance to water	0.01
Elevation	0.00
Farmland fragmentation	0.00

## Data Availability

None.
